# 

**Published:** 1948

**Authors:** 


### CONTENTS

| | Page |
|---|---|
| **Medicine and the Law** | |
| By CHARLES CORFIELD, M.D. | 97 |
| **Tasks ahead in Public Health** | |
| By PROFESSOR R. H. PARRY, M.D., F.R.C.P., D.P.H. | 102 |
| **Some Experiences in the Treatment of Arterial Injuries.** | |
| By J. J. MASON BROWN, *O.B.E.*, M.B., Ch.B. | 105 |
| **Clinical Note—Folic Acid** | 110 |
| **Surgical Treatment in Hypertension.** | |
| By HORACE EVANS, F.R.C.P. | 112 |
| **Editorial Notes** | 113 |
| **Reviews of Books** | 115 |
| **Meetings of Societies** | 117 |
| **Local Medical Notes** | 117 |

